# Minimally invasive percutaneous cannulated screw fixation for pelvic fractures: a retrospective case cohort study of clinical and radiological outcomes

**DOI:** 10.3389/fmed.2026.1807439

**Published:** 2026-06-19

**Authors:** Xichun Wang, Bin Hu, Wenjie Chen, Kunqiang Chen, Yi Cheng, Hongzhi Yang

**Affiliations:** 1 Department of Orthopedics, Jiujiang First People’s Hospital, Jiujiang, Jiangxi, China; 2 Jiujiang City Key Laboratory of Cell Therapy, Jiujiang, Jiangxi, China; 3 Department of Oncology, Ningguo People’s Hospital, Ningguo, Anhui, China

**Keywords:** clinical outcome, minimally invasive surgery, pelvic fracture, percutaneous cannulated screw fixation, radiological evaluation, Tile classification

## Abstract

**Objective:**

To describe the clinical and radiological outcomes of minimally invasive percutaneous cannulated screw fixation (MIPCSF) in a consecutive series of patients with unstable Tile B/C pelvic fractures, and to explore associations between Tile classification, surgical timing, and outcomes.

**Methods:**

A single-center retrospective cohort study was conducted on 248 consecutive patients with unstable Tile B/C pelvic fractures who underwent MIPCSF. Fracture reduction and screw placement accuracy were assessed via Matta scoring and Gertzbein-Robbins classification. Functional outcomes were evaluated using Majeed score, SF-36 and VAS. Subgroup analyses were performed by Tile classification (B vs. C) and surgical timing (≤72 h vs. >72 h).

**Results:**

Of 248 patients, 63.0% were male (mean age 42.8 ± 10.5 years) and 75.0% underwent early surgery. Mean intraoperative blood loss and operative time were 52.8 ± 18.6 mL and 68.5 ± 12.3 min. The excellent/good rate of Matta scoring was 92.3%, and clinically acceptable screw placement was 96.8%. Mean fracture healing time was 12.8 ± 3.2 weeks (healing rate 98.4%), with 18.5% developing degenerative changes. At final follow-up, mean Majeed score was 86.4 ± 10.2 (excellent/good rate 86.7%) and mean VAS was 1.8 ± 0.9. Tile C patients had longer operative time, more screws per patient, longer healing time, higher SI joint degeneration rate, and inferior functional scores than Tile B (all P-sub < 0.05 after Bonferroni correction). Early surgery was associated with shorter healing time (P-sub < 0.001) and lower SI joint degeneration (P-sub = 0.018) than delayed surgery. Matta excellent/good rate did not differ significantly between groups after correction for multiple comparisons.

**Conclusion:**

In this retrospective cohort, MIPCSF was associated with favorable clinical and radiological outcomes. Earlier surgical timing (≤72 h) was associated with shorter healing time and lower rates of SI joint degeneration. Tile C fractures were associated with longer operative times, more screws per patient, longer healing time, higher SI joint degeneration rates, and lower functional scores compared with Tile B fractures.

## 1 Introduction

Pelvic fractures account for 3–8% of all fractures, with an annual incidence of 20–37 per 100,000 population (1). High-energy mechanisms (traffic accidents, falls from height) predominate in younger adults, whereas fragility fractures now comprise up to 20% of cases in aging populations (2, 3). The complex pelvic anatomy—adjacent to visceral organs, iliac vessels, and the lumbosacral plexus—predisposes to severe hemorrhage and neurological injury (4). Hemodynamically unstable pelvic fractures are associated with substantial mortality, and the prompt application of appropriate hemorrhage control methods is essential to reduce acute hemorrhage-related deaths (5). Traditional open reduction and internal fixation (ORIF) enables direct fracture visualization and rigid stabilization but requires extensile exposures, incurring substantial blood loss, prolonged operative times, and increased risk of complications such as infection and iatrogenic nerve injury (6). Minimally invasive percutaneous cannulated screw fixation (MIPCSF) has emerged as an alternative, leveraging fluoroscopic or navigated guidance to achieve fixation through percutaneous incisions, thereby reducing soft-tissue morbidity and enabling early mobilization (7, 8). Technological refinements have progressively enhanced MIPCSF safety: 3D navigation reduces screw malposition rates compared with conventional fluoroscopy, while patient-specific 3D-printed guides facilitate preoperative planning in complex deformities (9, 10). Current evidence supporting MIPCSF remains limited by methodological heterogeneity: systematic reviews indicate small cohort sizes, short follow-up durations, and inconsistent functional endpoints (11). Specifically, the relationship between immediate reduction quality and long-term functional outcomes remains contentious. While anatomical reduction (≤4 mm displacement) correlates with superior radiological outcomes in some series, functional recovery may be acceptable with moderate residual displacement in select subsets (12). The influence of pelvic instability grade and surgical timing on union rates and post-traumatic degenerative changes remains poorly defined, with retrospective data yielding conflicting conclusions (13, 14). These uncertainties highlight the need for well-described clinical cohorts to inform patient selection and surgical planning.

This retrospective case cohort study describes outcomes from 248 consecutive patients undergoing MIPCSF for unstable pelvic fractures (Tile B/C), with minimum 24-month follow-up. While MIPCSF outcomes have been reported in smaller series, this study contributes to existing evidence by: (i) providing a large, homogeneous cohort with standardized long-term follow-up; (ii) exploring associations between early surgery and joint-specific outcomes; and (iii) applying methodologically rigorous analyses with appropriate correction for multiple comparisons. Using standardized radiological assessment (Matta scoring, Gertzbein-Robbins classification) and validated functional instruments (Majeed, SF-36), we aimed to: (1) describe benchmark clinical and radiological outcomes for MIPCSF in our institutional experience; (2) examine associations between Tile classification, surgical timing (≤72 h vs. >72 h), and joint-specific healing and degenerative changes; and (3) identify factors associated with post-traumatic morbidity, which may inform surgical decision-making and long-term monitoring protocols.

## 2 Materials and methods

### 2.1 Study design

This was a single-center retrospective cohort study conducted at our hospital from January 2020 to January 2024, with a minimum follow-up duration of 24 months to ensure the collection of mid-term radiological and functional outcomes. All research procedures adhered to the Declaration of Helsinki, and the study was exempted by the Institutional Review Board (IRB approval number: JJSDYRMYY-YXLL-2026-013). The requirement for informed consent was waived because the investigation used de-identified data collected during routine clinical care.

### 2.2 Inclusion and exclusion criteria

Patients were eligible for enrollment if they met the following inclusion criteria: aged between 18 and 65 years, diagnosed with unstable pelvic fractures classified as Tile type B or C (15), treated with minimally invasive cannulated screw internal fixation, followed up for at least 24 months, and had complete preoperative computed tomography (CT) scans, immediate postoperative imaging data, and serial follow-up radiological records. Patients were excluded if they had open fractures (Gustilo-Anderson type III), pathological fractures secondary to tumors, infections, or metabolic bone diseases, concurrent severe traumatic brain injury with a Glasgow Coma Scale score <8, a history of previous pelvic surgery, or were lost to follow-up or had incomplete clinical and radiological data.

### 2.3 Patient data

Relevant baseline data were extracted from the electronic medical records and imaging archives of the enrolled patients. Demographic characteristics including age, gender, and body mass index (BMI) were collected, along with details of injury mechanisms such as traffic accidents, falls from height, and crush injuries. Fracture classifications were recorded based on the Tile and Young-Burgess systems, while associated injuries were evaluated using the Injury Severity Score (ISS), with concurrent injuries involving the head, chest, abdomen, or extremities documented in detail. Surgical timing, defined as the interval from injury to operation, was retrieved as a potential confounding variable and the enrolled patients were further categorized into an early surgery group (≤72 h) and a delayed surgery group (>72 h) for subsequent subgroup analysis. The 72-h cutoff was selected because unstable pelvic fractures frequently require initial resuscitation and multidisciplinary clearance, making shorter windows impractical (16, 17). Comorbidities were recorded and quantified using the Charlson Comorbidity Index (CCI). Associated injuries were categorized by anatomical region (head, chest, abdomen, extremities) and quantified using the Injury Severity Score (ISS).

### 2.4 Surgical technique

#### 2.4.1 Preoperative planning

All patients underwent preoperative thin-slice CT scanning of the pelvis with a slice thickness of 1–2 mm, and three-dimensional (3D) reconstruction was performed using 3D Slicer (Version 4.13.0) or Mimics (Version 21.0) software to visualize the fracture morphology, pelvic ring stability, and anatomical landmarks. The entry point, direction, length, and diameter of cannulated screws were precisely planned based on the 3D reconstructed models, with special attention to avoiding neurovascular structures such as the sacral nerve foramina and iliac vessels. For complex fracture patterns, patient-specific navigation templates were designed and 3D printed to enhance the accuracy of screw placement, and the templates were sterilized prior to surgery for intraoperative application.

#### 2.4.2 Surgical procedure

Anesthesia was administered as either general anesthesia or combined spinal-epidural anesthesia, and patient positioning was determined based on the fracture type—supine position was used for anterior pelvic ring injuries, while the floating position (combination of supine and lateral decubitus) was adopted for posterior pelvic ring instability. The surgical procedure began with percutaneous sacroiliac joint screw placement: the entry point for S1 screws was located 1–2 cm below the posterior superior iliac spine, and the screw was advanced under fluoroscopic guidance towards the center of the sacroiliac joint, parallel to the sacral endplate, to ensure placement within the safe corridor and avoid breaching the sacral nerve foramina. For S2 screws, the entry point was 2–3 cm inferior to the S1 entry point, with a slightly more caudal direction to accommodate the anatomical structure of the S2 vertebra. Percutaneous pubic ramus screw fixation was performed either in an antegrade or retrograde manner: antegrade screws were inserted from the lateral aspect of the pubic tubercle, directed towards the ischiopubic ramus, while retrograde screws were placed from the inferior aspect of the pubic symphysis, advancing laterally along the pubic ramus. Anterior ring fixation—minimally invasive approach for all patients: All anterior pelvic ring injuries were treated using minimally invasive techniques. No patient required formal open reduction with extensile exposure. Supra-acetabular iliac screws were inserted when additional pelvic ring stability was required, with the entry point at the anterior superior iliac spine, directed posteromedially along the iliac fossa to engage the posterior iliac cortex. Intraoperative fluoroscopy was performed in anteroposterior, inlet, outlet, and oblique views to confirm optimal fracture reduction and screw position, ensuring no neurovascular compromise. Postoperatively, a drainage tube was placed in the surgical area if the estimated blood loss exceeded 200 mL, and standardized protocols were implemented: intravenous antibiotics were administered for 24–48 h for infection prophylaxis, and low-molecular-weight heparin was given for 10–14 days to prevent venous thromboembolism, with early mobilization encouraged as tolerated.

### 2.5 Data collection and outcome measures

#### 2.5.1 Primary outcome measures

Fracture reduction quality was assessed using immediate postoperative CT scans with the Matta scoring system, where scores were categorized as excellent (≤1 mm residual displacement), good (2–4 mm), fair (5–10 mm), and poor (>10 mm). Residual vertical and rotational displacements were measured in millimeters on coronal and axial CT images, respectively, using image analysis software. Screw position accuracy was evaluated according to the Gertzbein-Robbins classification: grade 1 indicated the screw was entirely within the bone, grade 2 denoted a breach of <2 mm, grade 3 a breach of 2–4 mm, and grade 4 a breach of >4 mm, with grades 1 and 2 considered clinically acceptable. Total fluoroscopy time, reduction-phase fluoroscopy time, and fixation-phase fluoroscopy time were recorded for each case. C-arm settings (kVp, mA, pulse rate) were standardized across all procedures. Fluoroscopy time recording: Total fluoroscopy time was prospectively recorded by the radiology technician and retrospectively separated into two phases: (i) reduction-phase fluoroscopy—from first fluoroscopic image to confirmation of satisfactory fracture reduction on AP/inlet/outlet views; and (ii) fixation-phase fluoroscopy, from first screw insertion to final screw position confirmation. This separation was performed by reviewing timestamped fluoroscopy logs and operative video records.

#### 2.5.2 Secondary outcome measures

For mid-term radiological outcomes, fracture healing time was determined as the time from surgery to the first radiological evidence of bridging callus on serial radiographs and CT scans, with nonunion defined as no signs of healing at 6 months postoperatively. Pelvic ring symmetry was assessed using the Mears criteria, which evaluate the alignment of the pelvic ring on anteroposterior and inlet/outlet radiographs. Degenerative changes were evaluated separately for the sacroiliac (SI) joints and hip joints based on follow-up imaging at 12, 18, and 24 months. SI joint degeneration was defined as the presence of any of the following on AP, inlet, and outlet pelvic radiographs and CT: (i) joint space narrowing <2 mm; (ii) subchondral sclerosis; (iii) subchondral cysts; (iv) osteophyte formation; or (v) ankylosis (18). These findings were graded using a modified Stäheli classification (Grade 0: none; Grade 1: mild, 1 finding; Grade 2: moderate, 2 findings; Grade 3: severe, ≥3 findings). Hip joint degeneration was defined as the presence of any of the following on AP and lateral hip radiographs: (i) joint space narrowing <2 mm in the superior weight-bearing zone; (ii) femoral head or acetabular osteophytes; (iii) subchondral sclerosis or cysts; or (iv) femoral head deformity (19). These were graded using the Kellgren-Lawrence classification (Grade 0–4), with Grade ≥2 considered degenerative.

The incidence of screw loosening or breakage was also documented through radiological review. Functional outcomes were evaluated using the Majeed pelvic function score (range 0–100, with ≥85 considered excellent, 70–84 good, 55–69 fair, and <55 poor), the SF-36 Health Survey (assessing eight domains: physical functioning, role-physical, bodily pain, general health, vitality, social functioning, role-emotional, and mental health), and the Visual Analog Scale (VAS) for pain (range 0–10, with 0 indicating no pain and 10 indicating the worst possible pain). Gait analysis was performed when feasible using a motion capture system to assess walking speed, step length, and symmetry. Complications were recorded throughout the follow-up period, including superficial or deep infection, neurovascular injury (assessed by clinical examination and electromyography if symptoms persisted), nonunion, malunion, and thromboembolic events such as deep vein thrombosis and pulmonary embolism.

### 2.6 Statistical analysis

Statistical analysis was performed using R 4.2.0, with a two-tailed *p*-value <0.05 considered statistically significant. Sample size calculation was based on preliminary data from a pilot study, with a significance level (*α*) of 0.05, power (1-*β*) of 0.8, and an effect size of 0.8 for the primary outcome, leading to a required sample size of 248 patients. Descriptive statistics were used to summarize continuous variables, which were presented as mean ± standard deviation (SD) if normally distributed (assessed by the Shapiro–Wilk test) or median with interquartile range (IQR) if non-normally distributed; categorical variables were expressed as frequencies and percentages. Between-group comparisons for continuous data were performed using the independent samples *t*-test (for normally distributed data) or the Mann–Whitney *U* test (for non-normally distributed data), while categorical data were compared using the *χ*^2^ test or Fisher’s exact test (when expected cell frequencies were <5).

Correlation analysis was conducted to explore the relationship between reduction quality (Matta score and residual displacement) and functional outcomes (Majeed score and VAS pain score) using Pearson’s correlation coefficient for normally distributed data or Spearman’s rank correlation coefficient for non-normally distributed data. Kaplan–Meier curves were constructed to estimate fracture healing time, and the log-rank test was used to compare healing times between different fracture types.

Multivariate Logistic regression analysis with a forward stepwise selection method was performed to identify independent risk factors for complications, with potential confounding variables including age, BMI, fracture type, ISS score, and surgical timing included in the initial model. Missing data were handled using multiple imputation with 10 imputed datasets, and sensitivity analysis was conducted to assess the robustness of the results by comparing outcomes from complete case analysis and multiple imputation. To address potential confounding in the surgical timing subgroup analysis, multivariate linear regression (for healing time) and logistic regression (for degenerative changes) were performed, adjusting for age, BMI, Tile classification, ISS, associated injuries, and reason for delayed surgery. Propensity score matching (1:1 nearest neighbor, caliper 0.1) was additionally performed to balance covariates between early and delayed surgery groups. Sensitivity analysis excluding transferred patients was conducted to assess robustness. To control for Type I error in subgroup analyses, the Bonferroni correction was applied. For Tile type B vs. C comparisons, the adjusted significance threshold was *α* = 0.0071 (0.05/7). For early vs. delayed surgery comparisons, the adjusted threshold was *α* = 0.025 (0.05/2). Additionally, the Benjamini-Hochberg false discovery rate (FDR) method (*q* = 0.05) was applied as a sensitivity analysis for the 7 Tile B vs. C comparisons. Both raw and adjusted *p*-values are reported. To assess whether surgical timing effects were independent of polytrauma burden, multivariate linear regression (for healing time and Majeed score) and multivariate logistic regression (for degenerative changes) were performed with the following covariates: age, BMI, Tile classification, ISS, associated injuries (head, chest, abdomen, extremities as separate binary variables), Charlson Comorbidity Index, and surgical timing (early vs. delayed). Subgroup analyses stratified by ISS (≤16 vs. >16) were additionally performed.

## 3 Results

### 3.1 Baseline characteristics of the study population

During the study period, 287 patients underwent MIPCSF for pelvic fractures. After applying inclusion and exclusion criteria, 39 patients were excluded: open fractures (*n* = 8), pathological fractures (*n* = 3), severe traumatic brain injury with Glasgow Coma Scale <8 (*n* = 5), history of previous pelvic surgery (*n* = 6), incomplete baseline data (*n* = 5), and failure to complete 24-month follow-up (*n* = 12). Ultimately, 248 patients met all eligibility criteria and were included in the final analysis. All 248 patients completed regular follow-up (median 28.6 ± 4.2 months, range 24–36 months). Their baseline characteristics were summarized in Table 1.

**Table 1:** Baseline demographic and clinical characteristics of the study population.

Characteristics	All patients (*n* = 248)
Age (years)	42.8 ± 10.5 (range, 18–65)
Gender, male *n* (%)	156 (63.0)
BMI (kg/m^2^)	24.3 ± 3.1
Injury mechanism, *n* (%)	—
Traffic accident	145 (58.5)
Fall from height	68 (27.4)
Crush injury	35 (14.1)
Young-Burgess type, *n* (%)	—
LC	102 (41.1)
APC	83 (33.5)
VS	63 (25.4)
Tile classification, *n* (%)	—
Type B	136 (54.8)
Type C	112 (45.2)
ISS, median (IQR)	16 (12–22)
Concurrent injuries, *n* (%)	78 (31.5)
Time to surgery (hours)	48.6 ± 18.3 (range, 24–120)
Early surgery (≤72 h), *n* (%)	186 (75.0)
>72 h (delayed)	62 (25.0)

Data are presented as mean ± standard deviation (SD), median (interquartile range [IQR]), or *n* (%). BMI, body mass index; ISS, Injury Severity Score; LC, lateral compression; APC, anteroposterior compression; VS, vertical shear.

Of the 248 patients, 156 were male (63.0%) and 92 female (37.0%), with mean age 42.8 ± 10.5 years (18–65 years) and mean BMI 24.3 ± 3.1 kg/m^2^. Traffic accidents (145 cases, 58.5%) were the main injury mechanism, followed by falls from height (68 cases, 27.4%) and crush injuries (35 cases, 14.1%). Tile classification: 136 (54.8%) type B, 112 (45.2%) type C. Young-Burgess classification: LC type (102 cases, 41.1%) predominated, followed by APC (83 cases, 33.5%) and VS types (63 cases, 25.4%). Median ISS was 16 (IQR 12–22); 78 patients (31.5%) had concurrent injuries (32 head, 26 chest, 20 abdomen). Mean time to surgery was 48.6 ± 18.3 h (24–120 h), with 186 (75.0%) undergoing early surgery (≤72 h) and 62 (25.0%) delayed surgery.

Baseline characteristics were comparable between early (≤72 h) and delayed (>72 h) surgery groups (Supplementary Table 1). There were no significant differences in fracture type distribution, ISS, associated injuries, or demographic variables (all *p* > 0.05). Among delayed surgery patients, the most common reason for delay was hemodynamic instability (45.2%), followed by inter-hospital transfer (29.0%) and resource constraints (25.8%).

### 3.2 Intraoperative outcomes

Intraoperative outcomes (operative efficiency, blood loss, screw placement, conversion to open surgery) were recorded, with detailed data in Table 2. All 248 patients completed minimally invasive surgery without conversion to open surgery. Mean operative time was 68.5 ± 12.3 min (47–123 min) and mean intraoperative blood loss 52.8 ± 18.6 mL (20–115 mL). Mean screws per patient were 3.2 ± 0.8 (2–5), total 783 screws (302 SI screws, 38.6%; 481 pubic ramus screws, 61.4%); 68 patients (27.4%) received supplementary supra-acetabular screws (mainly Tile type C). Total fluoroscopy time was 20.8 ± 4.8 min per case (12–36), comprising reduction-phase fluoroscopy (9.4 ± 3.5 min) and fixation-phase fluoroscopy (11.4 ± 3.1 min). Tile C fractures required significantly longer total fluoroscopy time than Tile B (24.6 ± 5.1 vs. 18.4 ± 4.2 min, *p* < 0.001). This difference was driven by reduction-phase fluoroscopy (Tile C: 12.3 ± 3.8 vs. Tile B: 7.1 ± 2.4 min, p < 0.001), while fixation-phase fluoroscopy showed no significant difference (12.3 ± 3.1 vs. 11.3 ± 2.8 min, *p* = 0.18). The number of fluoroscopic images per case was 14.2 ± 3.5 (10–26). No intraoperative complications occurred.

**Table 2:** Intraoperative outcomes of all patients undergoing minimally invasive cannulated screw internal fixation.

Intraoperative outcomes	All patients (*n* = 248)	Tile B (*n* = 136)	Tile C (*n* = 112)	*p*-value
Operative time (minutes)	68.5 ± 12.3 (47–123)	63.1 ± 10.5	75.3 ± 11.8	<0.001
Intraoperative blood loss (mL)	52.8 ± 18.6 (20–115)	51.2 ± 17.5	55.1 ± 19.8	0.42
Number of screws per patient	3.2 ± 0.8 (2–5)	2.9 ± 0.7	3.6 ± 0.7	<0.001
Total fluoroscopy time (minutes)	20.8 ± 4.8 (12–36)	18.4 ± 4.2	24.6 ± 5.1	<0.001
Reduction-phase fluoroscopy (minutes)	9.4 ± 3.5 (4–18)	7.1 ± 2.4	12.3 ± 3.8	<0.001
Fixation-phase fluoroscopy (minutes)	11.4 ± 3.1 (6–20)	11.3 ± 2.8	12.3 ± 3.1	0.18
Fluoroscopy times per case (number of images)	14.2 ± 3.5 (10–26)	13.1 ± 3.2	15.8 ± 3.6	<0.001
Screw placement time per screw (minutes)	11.3 ± 2.4	10.9 ± 2.1	11.8 ± 2.7	0.08
Supra-acetabular screws, *n* (%)	68 (27.4)	22 (16.2)	46 (41.1)	<0.001
SI screws, *n* (% of total)	302 (38.6)	168 (38.5)	134 (38.7)	0.96
Pubic ramus screws, *n* (% of total)	481 (61.4)	268 (61.5)	213 (61.3)	0.96
Conversion to open surgery, *n* (%)	0 (0.0)	0 (0.0)	0 (0.0)	—

Data are presented as mean ± SD (range) or *n* (%). *p*-values from independent *t*-test or *χ*^2^ test.

### 3.3 Radiological outcomes

#### 3.3.1 Fracture reduction quality

**Figure 1:**
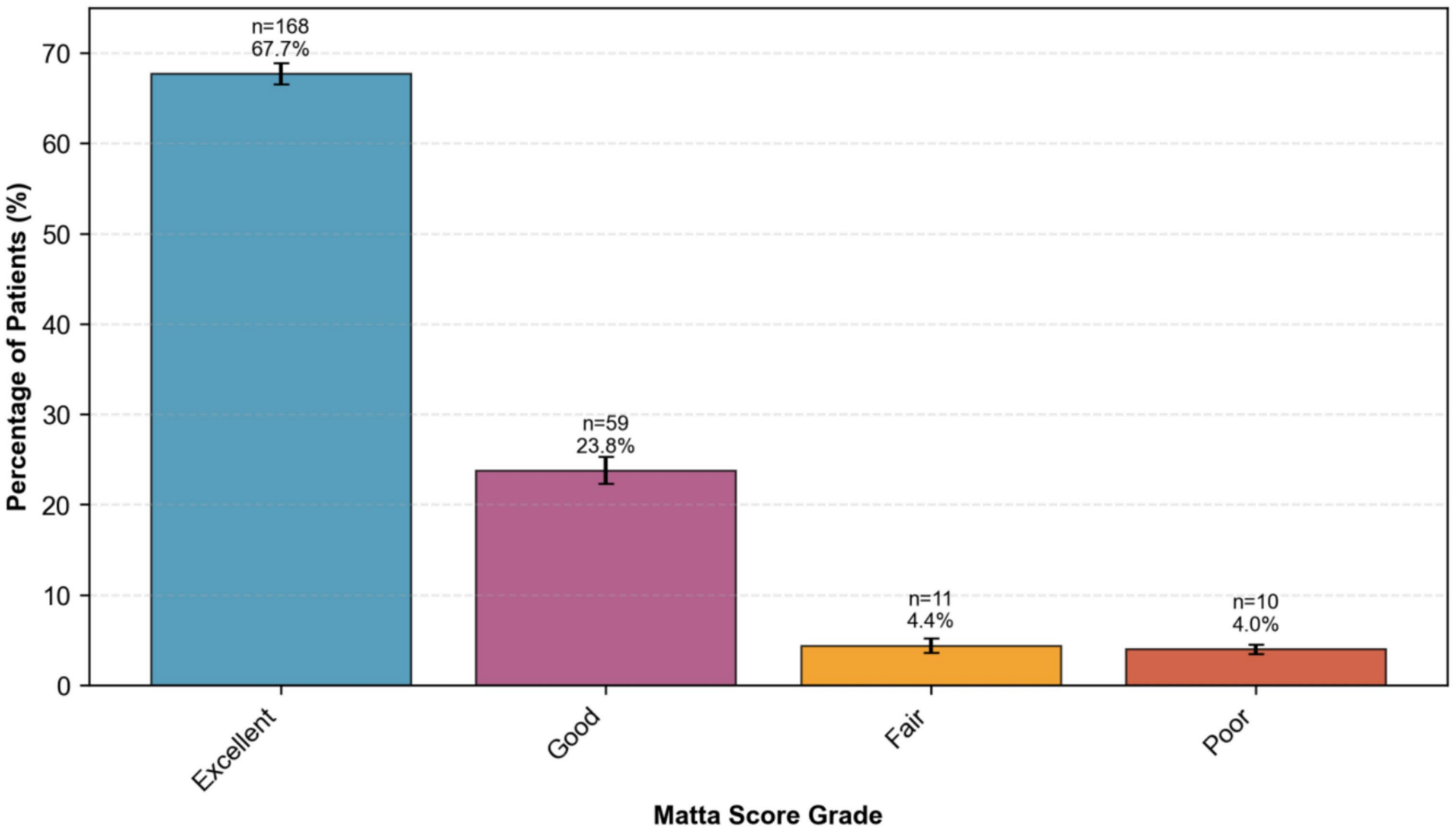
Fracture reduction quality assessed by Matta scoring system. Bar chart showing the distribution of Matta scores; error bars represent mean ± standard deviation (SD), and each bar is labeled with the corresponding sample size.

Immediate postoperative CT showed mean residual vertical displacement 3.2 ± 1.8 mm (0–12 mm) and rotational displacement 2.8 ± 1.5 mm (0–10 mm). By Matta scoring, 168 (67.7%) achieved excellent reduction, 59 (23.8%) good, 11 (4.4%) fair, 10 (4.0%) poor, with excellent/good rate 92.3%. Interobserver agreement for Matta scoring was high (*k* = 0.87, *p* < 0.001). Matta score distribution images are shown in Figure 1.

#### 3.3.2 Screw position accuracy

**Figure 2:**
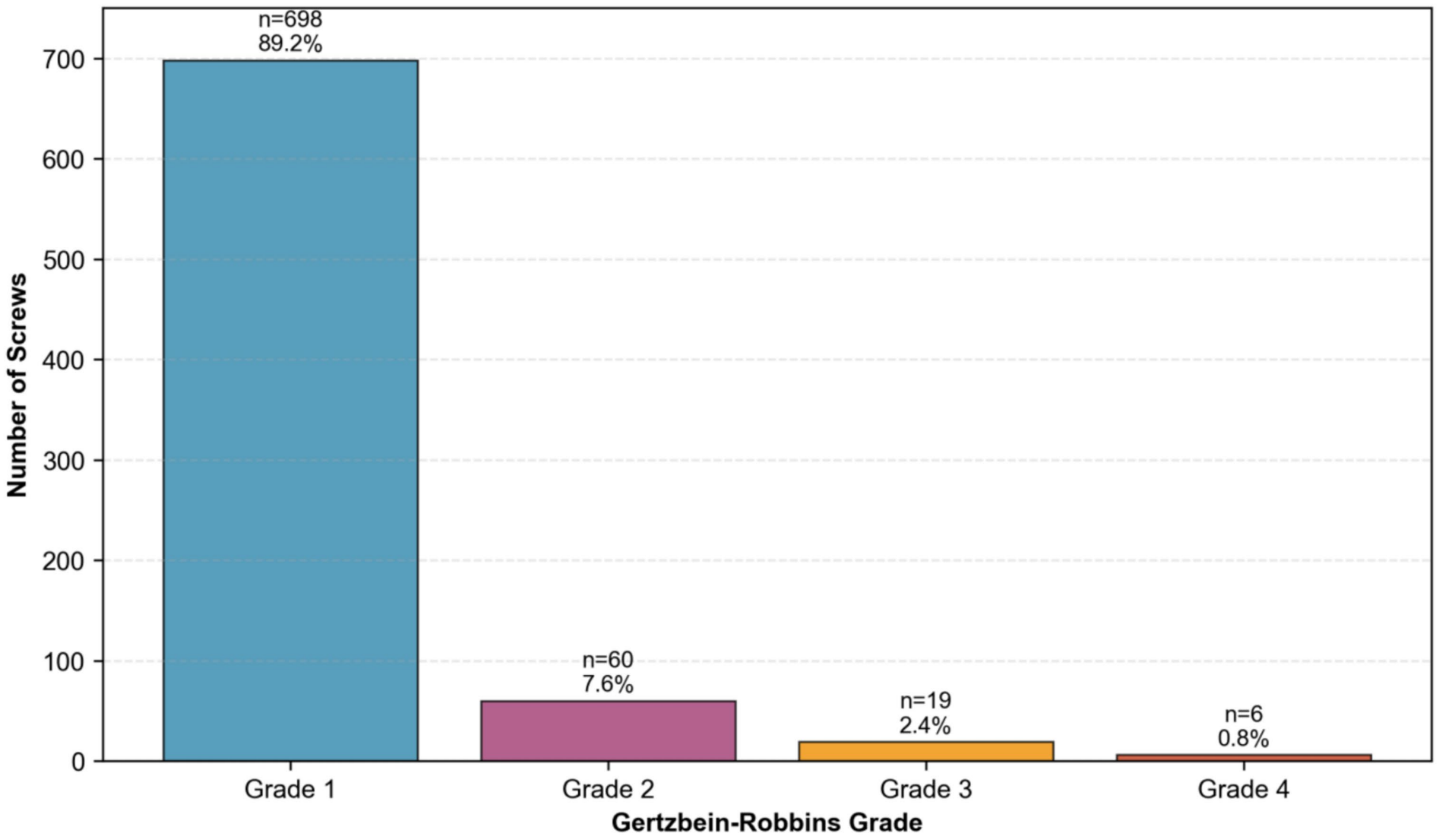
Screw position accuracy assessed by Gertzbein-Robbins classification. CT axial image showing grade 1 screw placement (entirely within bone) in S1; each image is labeled with the corresponding sample size for the respective grade (grade 1: *n* = 698 screws, 89.2%; grade 2: *n* = 60 screws, 7.6%) and the total number of screws implanted (*n* = 783).

By Gertzbein-Robbins classification, 698 (89.2%) screws were grade 1, 60 (7.6%) grade 2, 19 (2.4%) grade 3, 6 (0.8%) grade 4, with clinically acceptable rate (grades 1 + 2) 96.8%. No neurovascular complications were associated with screw breaches; interobserver agreement was good (*k* = 0.82, *p* < 0.001). Screw position grades are in Figure 2.

#### 3.3.3 Mid-term radiological follow-up

**Figure 3:**
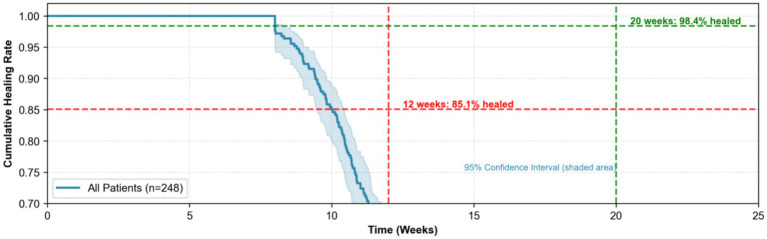
Kaplan–Meier curve for fracture healing time in all patients. The curve shows the cumulative healing rate over time, with a mean healing time of 12.8 ± 3.2 weeks (95% CI: 12.2–13.4). The curve is labeled with the total sample size (*n* = 248) and 95% confidence intervals (CIs) as statistical error annotations. The cumulative healing rate was 85.1% at 12 weeks and 98.4% at 20 weeks postoperatively.

All 248 patients achieved radiological fracture healing (mean time 12.8 ± 3.2 weeks, 8–20 weeks), with healing rate 98.4%; 4 patients (1.6%) had nonunion, successfully managed with revision surgery. Kaplan–Meier curve (Figure 3) showed 85.1% healing within 12 weeks and 98.4% within 20 weeks. Pelvic symmetry excellent/good rate was 93.5% by Mears criteria at last follow-up. Degenerative changes were analyzed separately by joint. SI joint degeneration occurred in 38 patients (15.3%), significantly more common in Tile C than Tile B fractures (22.3% vs. 9.6%, P_adj = 0.042 after Bonferroni correction) and in delayed vs. early surgery (24.2% vs. 12.4%, P_adj = 0.018). Hip joint degeneration was observed in 12 patients (4.8%), with no significant difference between Tile B and C (3.7% vs. 6.3%, *p* = 0.32) or early vs. delayed surgery (4.3% vs. 6.5%, *p* = 0.48). Four patients (1.6%) showed degeneration in both joints. Poor reduction quality (Matta fair/poor) was associated with higher SI joint degeneration (28.6% vs. 13.8%, *p* = 0.017) but not hip joint degeneration (9.5% vs. 4.2%, *p* = 0.21). Screw loosening/breakage occurred in 3 patients (1.2% of total cohort, 2.7% of Tile C), managed conservatively with no instability; no other radiological complications were reported (Table 3).

**Table 3:** Mid-term radiological outcomes of all patients during follow-up.

Radiological outcomes	All patients (*n* = 248)	Tile B (*n* = 136)	Tile C (*n* = 112)	*p*-value	P-adjust
Healing time (weeks)	12.8 ± 3.2 (8–20)	11.6 ± 2.8	14.2 ± 3.5	<0.001	<0.001*
Matta score excellent/good, *n* (%)	227 (92.3)	130 (95.6)	99 (88.4)	0.024	0.168
Residual vertical displacement (mm)	3.2 ± 1.8 (0–12)	—	—	—	—
Residual rotational displacement (mm)	2.8 ± 1.5 (0–10)	—	—	—	—
Screw placement acceptable rate (grade 1 + 2), *n* (%)	758 (96.8)	—	—	—	—
Pelvic symmetry excellent/good, *n* (%)	232 (93.5)	—	—	—	—
SI joint degeneration, *n* (%)	38 (15.3)	13 (9.6)	25 (22.3)	0.006	0.042^†,*^
Hip joint degeneration, *n* (%)	12 (4.8)	5 (3.7)	7 (6.3)	0.32	NS
Both SI and hip degeneration, *n* (%)	4 (1.6)	2 (1.5)	2 (1.8)	0.85	NS
Screw loosening/breakage, *n* (%)	3 (1.2)	0 (0.0)	3 (2.7)	0.032	0.224
Nonunion, *n* (%)	4 (1.6)	—	—	—	—

*Significant after Bonferroni correction for 7 Tile B vs. C comparisons (*α* = 0.0071). ^†^Significant after FDR correction (*q* = 0.05). “—” indicates not compared.

### 3.4 Functional outcomes

#### 3.4.1 Majeed pelvic function score

**Figure 4:**
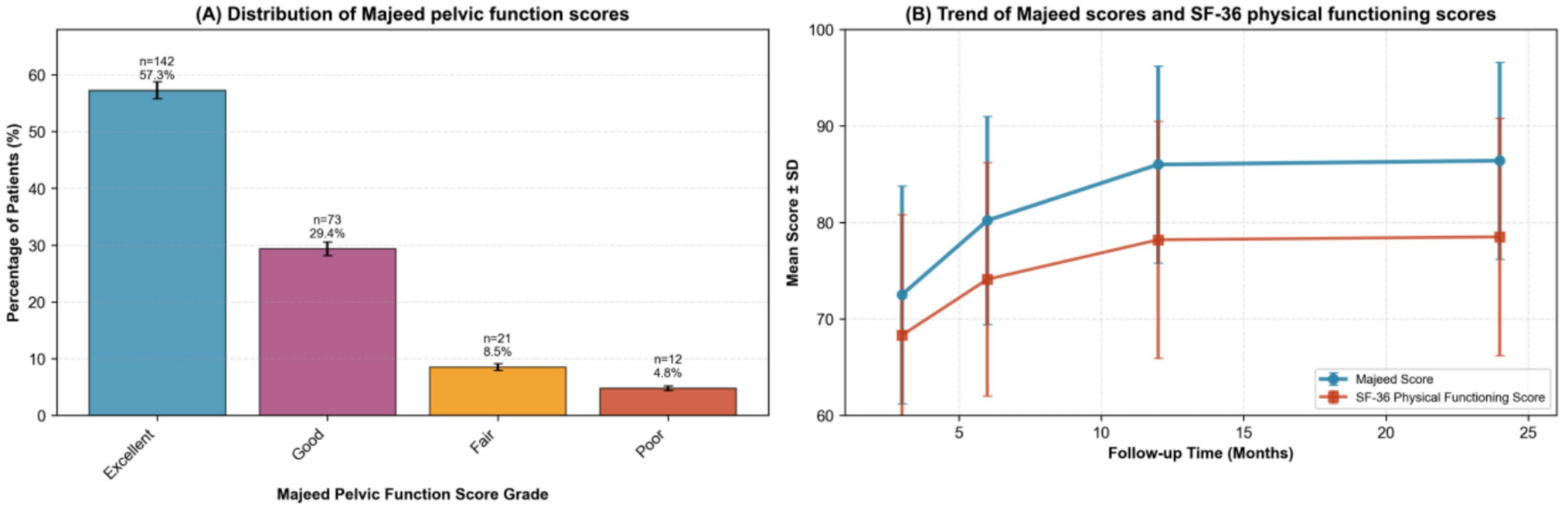
Functional outcomes at the last follow-up and over time. **(A)** Bar chart showing the distribution of Majeed pelvic function scores (excellent: *n* = 142, 57.3%; good: *n* = 73, 29.4%; fair: *n* = 21, 8.5%; poor: *n* = 12, 4.8%); **(B)** Line chart showing the trend of Majeed scores and SF-36 physical functioning scores from 3 months to 24 months postoperatively.

At the final follow-up, the mean Majeed pelvic function score of the patients was 86.4 ± 10.2 (range: 52–100), with an excellent/good rate of 86.7%. According to the Majeed scoring criteria, 142 patients (57.3%) achieved excellent function, 73 patients (29.4%) achieved good function, 21 patients (8.5%) achieved fair function, and 12 patients (4.8%) had poor function. The distribution of Majeed scores is shown in Figure 4A. Correlation analysis revealed a moderate positive association between Matta score category and Majeed score (Pearson *r* = 0.52, 95% CI: 0.42–0.61, *p* < 0.001). Patients with excellent/good reduction had higher mean Majeed scores than those with fair/poor reduction (90.3 ± 7.8 vs. 68.5 ± 9.2, *p* < 0.001).

#### 3.4.2 SF-36 health survey and VAS pain score

At the final follow-up, the mean scores of each domain of the SF-36 Health Survey were as follows: physical functioning 78.5 ± 12.3, role-physical 72.4 ± 15.6, bodily pain 76.8 ± 13.2, general health 74.3 ± 14.5, vitality 79.2 ± 11.8, social functioning 82.6 ± 10.7, role-emotional 80.5 ± 12.4, and mental health 83.4 ± 9.6. The mean VAS pain score was 1.8 ± 0.9 (range: 0–4). Patients with degenerative changes had slightly higher VAS scores than those without (2.3 ± 0.8 vs. 1.6 ± 0.8, *p* = 0.061), but the difference had no clinical significance. The Majeed score and SF-36 physical functioning score showed a gradual upward trend from 3 to 24 months postoperatively and stabilized after 12 months (Figure 4B). Residual vertical displacement showed a weak positive correlation with VAS pain score (Spearman *ρ* = 0.31, 95% CI: 0.19–0.42, *p* < 0.001).

### 3.5 Subgroup analysis of outcomes by tile classification and surgical timing

Subgroup analyses were performed by Tile classification (B vs. C) and surgical timing (≤72 h vs. >72 h), with Bonferroni correction applied to control Type I error (Table 4).

**Table 4:** Subgroup analysis of outcomes by Tile classification and surgical timing.

Outcome indicators	Tile Type B (*n* = 136)	Tile Type C (*n* = 112)	Raw *p*-value	P-sub	Early surgery (≤72 h, *n* = 186)	Delayed surgery (> 72 h, *n* = 62)	Raw *p*-value	P-sub
Operative time (minutes)	63.1 ± 10.5	75.3 ± 11.8	<0.001	<0.001*	67.2 ± 12.1	72.5 ± 13.4	0.12	0.35
Number of screws per patient	2.9 ± 0.7	3.6 ± 0.7	<0.001	<0.001*	—	—	—	—
Healing time (weeks)	11.6 ± 2.8	14.2 ± 3.5	<0.001	<0.001*	12.1 ± 2.9	14.7 ± 3.4	<0.001	<0.001*
Matta excellent/good rate, *n* (%)	130 (95.6)	99 (88.4)	0.024	0.168	173 (93.0)	56 (90.3)	0.08	0.24
SI joint degeneration, *n* (%)	13 (9.6)	25 (22.3)	0.006	0.042*^,^†	23 (12.4)	15 (24.2)	0.006	0.018*
Hip joint degeneration, *n* (%)	5 (3.7)	7 (6.3)	0.32	0.46	8 (4.3)	4 (6.5)	0.48	0.88
Screw loosening rate, *n* (%)	0 (0.0)	3 (2.7)	0.032	0.224	1 (0.5)	2 (3.2)	0.38	0.51
Majeed score (last follow-up)	89.8 ± 8.7	81.2 ± 10.5	<0.001	<0.001*	87.1 ± 9.8	84.2 ± 11.5	0.06	0.15
Intraoperative blood loss (mL)	51.2 ± 17.5	55.1 ± 19.8	0.42	NS	51.8 ± 18.2	55.6 ± 19.5	0.34	0.44
Nonunion rate, *n* (%)	2 (1.5)	2 (1.8)	0.65	0.77	—	—	—	—

*Significant after Bonferroni correction. ^†^Significant after FDR correction (*q* = 0.05). “—” indicates not compared.

Tile classification (B vs. C): After Bonferroni correction, Tile type C patients (*n* = 112) had significantly worse outcomes than type B (*n* = 136) in operative time (75.3 ± 11.8 vs. 63.1 ± 10.5 min, *p* < 0.001, *P*-sub < 0.001), number of screws per patient (3.6 ± 0.7 vs. 2.9 ± 0.7, *p* < 0.001, *P*-sub < 0.001), healing time (14.2 ± 3.5 vs. 11.6 ± 2.8 weeks, *p* < 0.001, *P*-sub < 0.001), SI joint degeneration (22.3% vs. 9.6%, *p* = 0.006, *P*-sub = 0.042), and Majeed score (81.2 ± 10.5 vs. 89.8 ± 8.7, *p* < 0.001, *P*-sub < 0.001). The Matta excellent/good rate (88.4% vs. 95.6%, *p* = 0.024, *P*-sub = 0.168) and screw loosening rate (2.7% vs. 0.0%, *p* = 0.032, *P*-sub = 0.224) did not remain significant after correction. No significant differences were observed in intraoperative blood loss (55.1 ± 19.8 vs. 51.2 ± 17.5 mL, *p* = 0.42), screw placement acceptable rate (96.2% vs. 97.1%, *p* = 0.78), nonunion rate (1.8% vs. 1.5%, *p* = 0.65), or hip joint degeneration (6.3% vs. 3.7%, *p* = 0.32).

Surgical timing (≤72 h vs. >72 h): After Bonferroni correction, early surgery (*n* = 186) was associated with shorter healing time (12.1 ± 2.9 vs. 14.7 ± 3.4 weeks, *p* < 0.001, *P*-sub < 0.001) and lower SI joint degeneration (12.4% vs. 24.2%, *p* = 0.006, *P*-sub = 0.018) than delayed surgery (*n* = 62). No significant differences were noted in operative time (67.2 ± 12.1 vs. 72.5 ± 13.4 min, *p* = 0.12), intraoperative blood loss (51.8 ± 18.2 vs. 55.6 ± 19.5 mL, *p* = 0.34), Matta excellent/good rate (93.0% vs. 90.3%, *p* = 0.08), Majeed score (87.1 ± 9.8 vs. 84.2 ± 11.5, *p* = 0.06), screw loosening rate (0.5% vs. 1.6%, *p* = 0.38), or hip joint degeneration (4.3% vs. 6.5%, *p* = 0.48).

After adjusting for ISS, associated injuries (head, chest, abdomen, extremities), Charlson Comorbidity Index, age, BMI, and Tile classification, early surgery remained independently associated with shorter healing time (adjusted *β* = −2.6, 95% CI: −4.1 to −1.1, *p* = 0.001) and lower SI joint degeneration (adjusted OR = 0.46, 95% CI: 0.24–0.88, *p* = 0.019). Associated injuries independently predicted lower Majeed scores (*β* = −4.5, 95% CI: −7.8 to −1.2, *p* = 0.008), but did not eliminate the independent effect of surgical timing (*β* = −2.9, 95% CI: −5.1 to −0.7, *p* = 0.010). In patients with ISS ≤ 16 (*n* = 150), early surgery was associated with shorter healing time (11.8 ± 2.6 vs. 14.2 ± 3.3 weeks, *p* = 0.002). In patients with ISS > 16 (*n* = 98), this association persisted (12.6 ± 3.2 vs. 15.4 ± 3.6 weeks, p = 0.008), indicating consistency across injury severity strata (Supplementary Table 2).

### 3.6 Complications

A total of 18 complications occurred in 16 patients (6.5%), no fatal cases: 4 nonunion (1.6%), 3 screw loosening (1.2%), 6 superficial surgical site infections (2.4%), 3 transient sciatic nerve irritation (1.2%), 2 deep venous thrombosis (0.8%). All complications were managed effectively with conservative or revision treatment. At last follow-up, all patients with complications recovered well without long-term sequelae.

## 4 Discussion

The present retrospective case cohort study describes the clinical outcomes, radiological findings, and factors associated with outcomes following minimally invasive cannulated screw internal fixation in 248 patients with unstable pelvic fractures, with a median follow-up of 28.6 months. In this single-arm series, MIPCSF was associated with minimal intraoperative blood loss, acceptable fracture reduction quality, accurate screw placement, and favorable functional recovery. These findings are consistent with published reports of minimally invasive orthopedic techniques and contribute observational data regarding MIPCSF in pelvic fracture management. However, in the absence of a concurrent control group (e.g., open reduction and internal fixation or non-operative management), no causal inferences regarding superiority over alternative strategies can be drawn.

Intraoperative outcomes are critical indicators of surgical feasibility and safety. In this study, the mean operative time was 68.5 ± 12.3 min, and the mean intraoperative blood loss was only 52.8 ± 18.6 mL, with no conversions to open surgery. Historical reports of traditional open reduction and internal fixation (ORIF) describe operative times exceeding 120 min and blood loss of 300–800 mL (20, 21). However, direct comparison is not possible given the lack of a concurrent control group in our study; published ORIF data derive from heterogeneous patient populations, surgical eras, and outcome assessment methodologies, and selection bias may favor less severely injured patients for minimally invasive approaches. The reduced invasiveness of MIPCSF—achieved through small percutaneous incisions and minimal soft tissue dissection—not only minimizes intraoperative trauma but also reduces the risk of neurovascular injury and postoperative infection (22, 23). The high clinically acceptable screw placement rate (96.8%) further validates the reliability of fluoroscopically guided screw implantation, supported by preoperative 3D reconstruction planning and, in complex cases, patient-specific navigation templates (24, 25). This precision is crucial given the pelvis’s anatomical complexity, as improper screw placement can lead to sacral nerve injury, vascular compromise, or implant failure. The disproportionate increase in reduction-phase fluoroscopy for Tile C fractures (73% increase vs. Tile B) highlights that reduction—not fixation—is the primary determinant of radiation exposure in complex pelvic fractures. This finding has practical implications: (i) surgeon training should prioritize efficient closed reduction techniques for Tile C fractures; (ii) 3D navigation or robotic assistance may be most cost-effective during the reduction phase rather than screw placement; and (iii) radiation protection measures (lead shielding, pulsed fluoroscopy) should be maximized during prolonged reduction maneuvers.

Radiological outcomes reflect the structural success of fracture fixation and long-term stability. The excellent/good rate of fracture reduction (92.3%) by Matta scoring and the high pelvic symmetry rate (93.5%) by Mears criteria suggest that MIPCSF was associated with restoration of pelvic ring anatomy in this cohort, which may be relevant to functional recovery (26). The mean fracture healing time of 12.8 ± 3.2 weeks and 98.4% healing rate are comparable to or better than those reported in previous studies (27), suggesting that MIPCSF provides sufficient stability to promote bone union (28). Notably, only 1.2% of patients experienced screw loosening/breakage, and 1.6% had nonunion—complication rates consistent with or lower than those reported in previous MIPCSF series (up to 4.5% hardware failure) (29). Direct comparison with ORIF complication rates is limited by the lack of a concurrent control group; published ORIF data from other anatomic regions (e.g., 8% implant failure in proximal humerus fractures) may not be generalizable to pelvic fractures due to differences in biomechanics, surgical approach, and patient population (30). The low overall incidence of degenerative changes (18.5%) supports the long-term structural preservation achieved with MIPCSF. Notably, when analyzed separately, SI joint degeneration (15.3%) was the predominant finding, while hip joint degeneration was uncommon (4.8%). This distribution likely reflects the primary load-bearing role of the SI joint in pelvic ring stability, whereas hip joint changes may represent pre-existing osteoarthritis or concomitant acetabular injury rather than direct consequences of pelvic ring instability.

Functional recovery is the ultimate goal of pelvic fracture treatment (31). At final follow-up, the mean Majeed score (86.4 ± 10.2) and excellent/good rate (86.7%) indicate that most patients in this cohort achieved satisfactory pelvic and lower limb function. The moderate positive correlation between fracture reduction quality (Matta score) and Majeed scores (r = 0.52, 95% CI: 0.42–0.61, *p* < 0.001) is consistent with an association between anatomical reduction and superior functional outcomes. This finding aligns with biomechanical principles suggesting that residual displacement may contribute to gait abnormalities, chronic pain, or post-traumatic arthritis (12, 32). However, we emphasize that this correlation does not prove causation. Residual confounding from unmeasured variables—such as muscle strength, psychological resilience, rehabilitation compliance, or pre-injury functional status—may independently influence both reduction quality and functional recovery. Prospective studies with standardized rehabilitation protocols are needed to infer a causal relationship. The low mean VAS pain score (1.8 ± 0.9) and favorable SF-36 domain scores confirm that MIPCSF effectively alleviates pain and improves health-related quality of life. This adequate pain relief, facilitated by minimal surgical trauma, allows early mobilization, which in turn prevents muscle atrophy, joint stiffness, and deep venous thrombosis (33, 34). Notably, deep venous thrombosis occurred in only 0.8% of patients in this study. While our data demonstrate a statistically significant association between anatomical reduction and functional outcomes, we caution against inferring causality from this retrospective observational design. Alternative explanations include reverse causation, confounding by indication, and unmeasured mediator variables. These limitations underscore the need for randomized trials comparing anatomical vs. acceptable reduction thresholds.

Subgroup analysis explored factors associated with differential outcomes. After Bonferroni correction for multiple comparisons, Tile Type C fractures—characterized by complete pelvic ring disruption and vertical instability—were associated with longer operative times, more implants, longer healing time, higher SI joint degeneration rates, and lower functional scores compared with Type B fractures. This is consistent with the greater anatomical complexity and instability of Type C injuries, which require more extensive fixation and precise reduction (35, 36). Notably, the Matta excellent/good rate (88.4% vs. 95.6%, P-sub = 0.168) and screw loosening rate (2.7% vs. 0.0%, P-sub = 0.224) did not remain significant after correction, suggesting that while Tile C fractures present greater technical challenges, the differences in reduction quality and hardware failure risk may be less pronounced than initially observed. Nevertheless, the significantly higher SI joint degeneration rate in Tile C patients (22.3% vs. 9.6%, P-sub = 0.042) underscores the importance of rigorous perioperative management, including prolonged protected weight-bearing and close radiological monitoring. Additionally, after Bonferroni correction, earlier surgery (≤72 h) was associated with shorter healing times and lower SI joint degeneration rates compared with delayed surgery (>72 h), while no significant differences were observed in operative time (*p* = 0.12), blood loss (*p* = 0.34), Matta score (*p* = 0.08), or Majeed score (*p* = 0.06). These associations are consistent with the “damage control orthopedics” principle, which posits that earlier fixation may stabilize the pelvic ring before fibrous callus consolidation and reduce secondary soft tissue injury (37, 38). However, because surgical timing was not randomly assigned, these associations may reflect confounding by indication (e.g., hemodynamically unstable patients requiring delayed surgery) (37, 38). A key consideration is whether delayed surgery reflects more severely injured polytrauma patients, rather than a protective effect of earlier fixation per se. In multivariate analysis adjusting for ISS, specific associated injuries, and comorbidities, earlier surgery remained associated with shorter healing time and lower SI joint degeneration. Notably, associated injuries were associated with inferior functional outcomes (lower Majeed scores) but did not fully account for the surgical timing association. These findings suggest that earlier pelvic fixation may provide biomechanical benefits—such as stabilizing the pelvic ring before fibrous callus formation and reducing shear stress on the SI joint—independent of general trauma burden. However, residual confounding from unmeasured factors cannot be excluded, and causality cannot be inferred from this observational design. The anatomical specificity—SI joint preservation without significant hip joint effect—suggests that early intervention primarily protects the load-bearing posterior pelvic ring rather than the hip joint itself. Delayed surgery may lead to fibrous callus formation, increased reduction difficulty, and prolonged instability, contributing to SI joint degeneration over time (39).

This study has several strengths, including a large sample size (*n* = 248), long-term follow-up (≥24 months), and comprehensive outcome assessment (intraoperative, radiological, functional, and safety). The subgroup analysis by Tile classification and surgical timing provides valuable insights into personalized treatment strategies. First and most fundamentally, this is a single-arm retrospective case cohort study without a concurrent control group (e.g., ORIF or non-operative management). All comparisons with alternative treatments rely on published historical data and should be interpreted cautiously due to inherent selection, temporal, assessment, and publication biases. The study design precludes any conclusions regarding the comparative efficacy or superiority of MIPCSF over alternative strategies. The observed associations between surgical timing, fracture type, and outcomes are hypothesis-generating and require validation in randomized controlled trials. Second, the study excluded patients with open fractures, pathological fractures, or severe traumatic brain injury, limiting generalizability to more complex patient populations; third, functional outcomes relied on patient-reported scores (Majeed, SF-36) and lacked objective measures such as muscle strength testing or gait analysis in all patients. Future research should integrate both subjective patient-reported outcomes and objective quantitative assessments like biomechanical gait analysis, isokinetic muscle strength testing, and radiological quantitative parameters to conduct a more comprehensive and in-depth evaluation of the therapeutic effect of MIPCSF for pelvic fractures. Fourth, the non-random allocation of surgical timing represents an inherent confounding bias. Delayed surgery was influenced by hemodynamic status, transfer logistics, and resource availability, which may independently affect prognosis. Although we adjusted for these factors using multivariate regression and propensity score matching, and conducted sensitivity analyses excluding transferred patients, residual confounding from unmeasured factors (e.g., surgeon experience, precise hemodynamic parameters) cannot be fully excluded in this retrospective design. The observed associations should be interpreted as hypothesis-generating rather than definitive evidence of causation. The observed association between reduction quality and functional outcomes is associative, not causal. Despite statistical significance, residual confounding from unmeasured variables cannot be excluded. Fifth, while we adjusted for associated injuries and comorbidities using multivariate regression, unmeasured confounders (e.g., precise hemodynamic parameters, transfusion requirements, or specific injury patterns within anatomical regions) may still influence outcomes. The observational design precludes definitive causal inference.

## 5 Conclusion

In this retrospective case cohort of 248 patients, MIPCSF was associated with acceptable clinical and radiological outcomes, including minimal intraoperative blood loss, satisfactory reduction quality, and favorable functional recovery. Earlier surgical timing (≤72 h) was associated with shorter fracture healing time and lower rates of SI joint degeneration. Tile Type C fractures were associated with longer operative times, more implants, longer healing time, higher SI joint degeneration rates, and lower functional scores compared with Tile B fractures, suggesting that these injuries warrant rigorous perioperative management. These observational findings require validation in prospective comparative studies. Future research should focus on prospective comparisons with ORIF, clinical validation of patient-specific navigation technologies for complex fractures, and long-term follow-up (≥5 years) to evaluate the durability of functional recovery and joint health.

## Data Availability

The original contributions presented in the study are included in the article/Supplementary material, further inquiries can be directed to the corresponding author.
