# Supplementary material for: Minimally invasive percutaneous cannulated screw fixation for pelvic fractures: a retrospective case cohort study of clinical and radiological outcomes
Source: Front Med (Lausanne). 2026 Jun 19;13:1807439. doi: 10.3389/fmed.2026.1807439 (PMC13328408; doi:10.3389/fmed.2026.1807439)
Supplement: Supplementary file 1 [file Table_1.DOCX]

**Supplementary Table 1**: Baseline Characteristics Comparison Between Early (≤72h) and Delayed (>72h) Surgery Groups

| Characteristics | Early Surgery  (≤72h, n=186) | Delayed Surgery  (>72h, n=62) | P-value |
| --- | --- | --- | --- |
| Age (years) | 42.5 ± 10.3 | 43.7 ± 11.1 | 0.44 |
| Gender, male n (%) | 118 (63.4) | 38 (61.3) | 0.76 |
| BMI (kg/m²) | 24.2 ± 3.0 | 24.6 ± 3.4 | 0.38 |
| Injury mechanism, n (%) |  |  | 0.82 |
| Traffic accident | 108 (58.1) | 37 (59.7) |  |
| Fall from height | 52 (28.0) | 16 (25.8) |  |
| Crush injury | 26 (14.0) | 9 (14.5) |  |
| Tile classification, n (%) |  |  | 0.78 |
| Type B | 101 (54.3) | 35 (56.5) |  |
| Type C | 85 (45.7) | 27 (43.5) |  |
| Young-Burgess type, n (%) |  |  | 0.71 |
| LC | 76 (40.9) | 26 (41.9) |  |
| APC | 63 (33.9) | 20 (32.3) |  |
| VS | 47 (25.3) | 16 (25.8) |  |
| ISS, median (IQR) | 16 (12–22) | 17 (13–23) | 0.42 |
| Concurrent injuries, n (%) | 57 (30.6) | 21 (33.9) | 0.56 |
| Head | 23 (12.4) | 9 (14.5) |  |
| Chest | 20 (10.8) | 6 (9.7) |  |
| Abdomen | 14 (7.5) | 6 (9.7) |  |
| Reason for delay, n (%) | — |  |  |
| Hemodynamic instability | — | 28 (45.2) |  |
| Inter-hospital transfer | — | 18 (29.0) |  |
| Resource constraints | — | 16 (25.8) |  |

Data are presented as mean ± SD, median (IQR), or n (%). P-values for continuous variables from independent t-test or Mann-Whitney U test; categorical variables from χ² or Fisher's exact test.

**Supplementary Table 2**: Multivariate Analysis of Factors Associated with Healing

Time and SI Joint Degeneration

| Variable | Healing Time  (β, 95% CI) | P-value | SI Degeneration  (OR, 95% CI) | P-value |
| --- | --- | --- | --- | --- |
| Early surgery (≤72h) | -2.6 (-4.1 to -1.1) | 0.001 | 0.46 (0.24–0.88) | 0.019 |
| Tile C (vs. B) | 2.4 (1.0 to 3.8) | 0.001 | 2.12 (1.15–3.91) | 0.015 |
| ISS (per point) | 0.08 (-0.02 to 0.18) | 0.12 | 1.03 (0.98–1.08) | 0.28 |
| Head injury | 0.6 (-0.8 to 2.0) | 0.38 | 1.25 (0.62–2.52) | 0.53 |
| Chest injury | 0.4 (-0.9 to 1.7) | 0.54 | 1.18 (0.58–2.40) | 0.65 |
| Abdomen injury | 0.3 (-1.1 to 1.7) | 0.68 | 1.08 (0.48–2.43) | 0.85 |
| Extremity injury | 0.2 (-1.0 to 1.4) | 0.74 | 0.95 (0.46–1.96) | 0.89 |
| CCI (per point) | 0.15 (-0.2 to 0.5) | 0.42 | 1.08 (0.82–1.42) | 0.58 |
| Age (per year) | 0.03 (-0.04 to 0.10) | 0.38 | 1.01 (0.98–1.04) | 0.52 |

R² = 0.28 for healing time model; Nagelkerke R² = 0.22 for degeneration model.
